# The hepatic steatosis, steatohepatitis and metabolic dysfunction: distinct roles in hepatocellular carcinoma occurrence in chronic hepatitis B patients

**DOI:** 10.1007/s12072-024-10675-5

**Published:** 2024-06-27

**Authors:** Xindan Hu, Ying Wen

**Affiliations:** Department of Infectious Diseases, The First Affiliated Hospital of China Medical University, No. 155, Nanjing North Street, Heping District, Shenyang, 110001 Liaoning China

Dear Editor,

We appreciated the valuable findings of the article reported by Huang et al. [1]. In this study, apart from some common important factors of predicting hepatocellular carcinoma (HCC) incidence, metabolic risk factors especially type 2 diabetes mellitus was the risk factor, while hepatic steatosis was the protective factor, whereas there are several key issues deserved to be further addressed.

First, although most studies suggest that HBV infection reduces the incidence of non-alcoholic fatty liver disease (NAFLD) than that in the general population [2], the global prevalence of steatosis in chronic viral hepatitis B (CHB) patients was still as high as 34.93%, and the concurrent metabolic factors as steatosis risky factor in CHB patients, which is identical to the general population. Moreover, different from persistent steatosis, patients with intermittent fatty liver should be grouped separately and provided detailed body weight change and metabolic improvement.

Second, many studies demonstrated a positive correlation between steatosis and liver fibrosis, especially the persistent severe hepatic steatosis. Nevertheless, several studies showed no correlation between steatosis and fibrosis. Importantly, a recent meta-analysis showed negative correlation between steatosis and cirrhosis. However, some studies highlighted the metabolic factors exacerbated the progression of liver fibrosis. Notably, most studies showed that metabolic dysfunction-associated steatohepatitis increased the risk of long-term mortality/transplantation and liver-related events in CHB patients [1, 3].

Third, some studies have shown that steatosis is positively correlated with the occurrence of HCC in CHB patients, while other studies did not find this correlation. Several recent studies implied that steatosis reduced the incidence of HCC in CHB patients receiving antiviral therapy [4] or in untreated CHB patients [1]. Importantly, non-alcoholic steatohepatitis (NASH) and the metabolic factors were significantly associated with an increased HCC risk [1]. Although there are inconclusive results for the association between metabolic dysfunction-associated fatty liver disease (MAFLD) and risk of HBV-related HCC, most studies showed metabolic dysfunction-associated steatohepatitis increased the HCC risk [3].

Lastly, the inconsistent results from heterogeneity studies using different steatosis diagnostic methods should be considered. The biopsy-proven steatosis-CHB studies presented an increased risk of HCC, and NASH as the risk factor of liver fibrosis [5] and poor prognosis. Nevertheless, in non-invasive methods as the routinely used in real-world practice, the inconclusive results highlighted their limitations and confounding factors. Moreover, the recruited patients in different studies should be distinguished as receiving antiviral therapy [4], untreated, and mixed. Although untreated CHB patients were recruited in this study, half patients receiving anti-HBV therapy before HCC development should be also entered into model analysis as well.

In conclusion, patients with steatosis should be detailed classified into simple steatosis and steatohepatitis with or without metabolic factors. Importantly, other steatosis etiologies should be carefully differentiated. The persistent undetectable HBV DNA, weight management and regular HCC screening should be recommended. The optimal HCC screening strategy or improving metabolic dysfunction strategy for reducing HCC incidence should be further investigated.

## Data Availability

Not applicable.
